# Anaplastic lymphoma kinase–positive anaplastic large cell lymphoma presenting as diffuse pulmonary infiltrates

**DOI:** 10.1002/ccr3.3010

**Published:** 2020-07-03

**Authors:** Shuku Sato, Yotaro Tamai

**Affiliations:** ^1^ Division of Hematology Shonan Kamakura General Hospital Kanagawa Japan

**Keywords:** anaplastic lymphoma kinase‐positive anaplastic large cell lymphoma, disseminated pulmonary lymphomatous

## Abstract

Anaplastic large cell lymphoma has a characteristic sinusoidal growth pattern that presents as pulmonary lymphangitic involvement causing respiratory distress. Recognition and prompt treatment of this entity can result in dramatic response to therapy.

## Graphical abstract

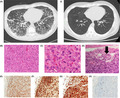

Anaplastic lymphoma kinase (ALK)–positive anaplastic large cell lymphoma (ALCL) exhibits a highly aggressive clinical course. Because of its sinusoidal growth, which is a characteristic pattern of ALCL, lymphomatous infiltration of the pulmonary lymphangitis occurs and causes severe respiratory distress.

A 29‐year‐old man presented with difficulty in breathing. His oxygen saturation was 80% on room air, and he required mechanical ventilation. Chest computed tomography revealed ground‐glass opacities, and thickened interlobular septa and pleura (Figure 1A). He had generalized lymphadenopathy and abdominal swelling secondary to splenomegaly. His lactate dehydrogenase level was normal (207 U/L), but the solved IL‐2 receptor level was high (57 022 U/mL). Inguinal lymph node biopsy confirmed the diagnosis of ALK‐positive anaplastic large cell lymphoma (ALCL) (Figure 1B‐H). The patient underwent chemotherapy (CHOP treatment), and his ventilatory parameters improved remarkably (Figure 1I). He was extubated 3 days after the initiation of chemotherapy.

**Figure 1:**
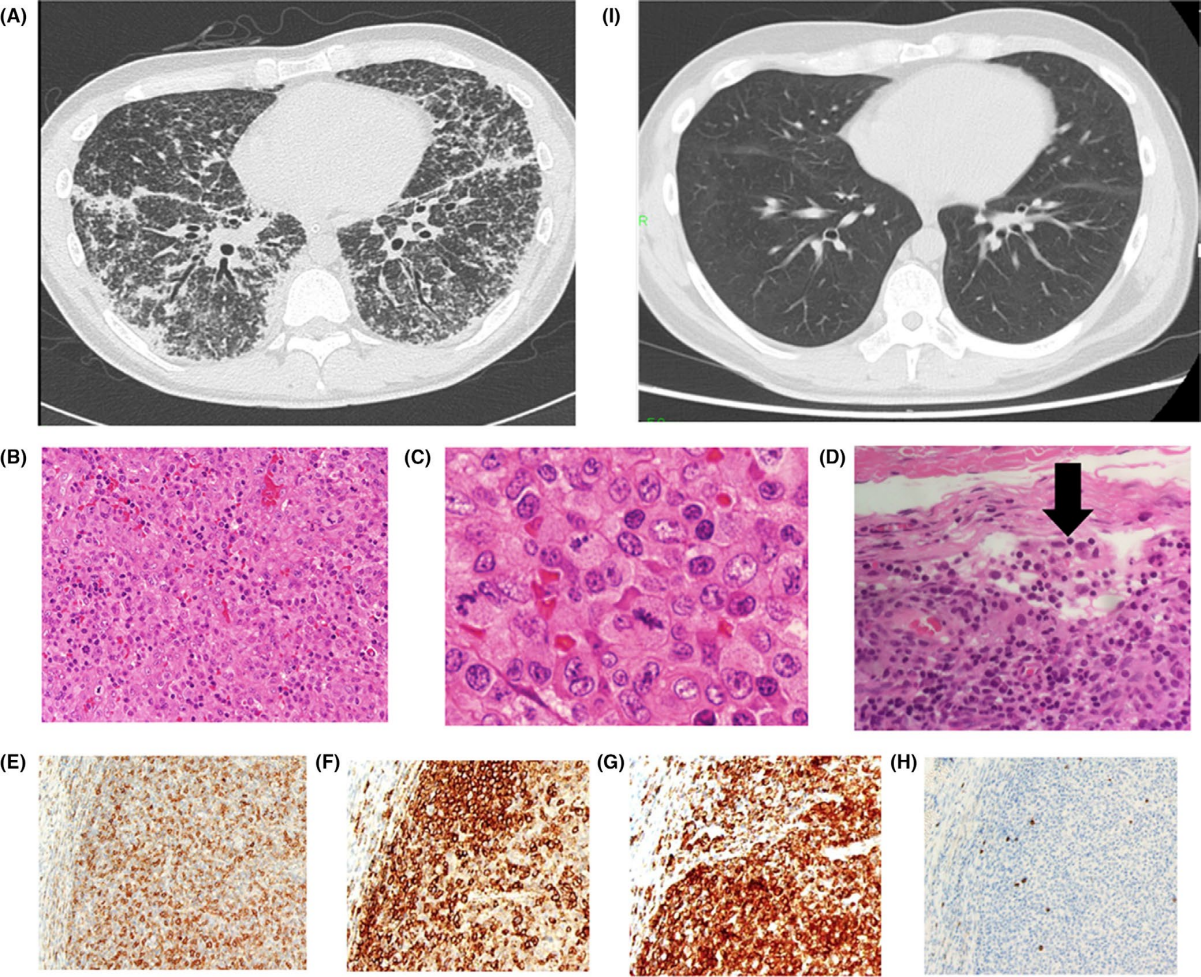
Chest computed tomography scan on admission showing nodular thickening of interlobular septa and pleura (A), and after chemotherapy, he achieved complete remission (i). Histological examination of lymph node biopsy showing neoplastic T cells with round‐ or bean‐shaped nuclei forming clusters and small sheets (hematoxylin and eosin, original magnifications ×20 (B), ×100 (C)) and sinusoidal involvement by anaplastic large cell lymphoma (D); immunohistological examination showing positive reactivity for CD4 (E), CD30 (G), and ALK (H), and negative reactivity for CD20 (F)

Lung involvement of ALCL occurs in 13% of pediatric patients because of dissemination.^1^ The frequency of lung involvement in adults is not known and is probably low. The characteristic pattern of ALCL in the lymph nodes is sinusoidal growth and perivascular distribution. Kinney et al^2^ reported that the guanine nucleotide exchange factors VAV3 and VAV1^2^ may be direct or indirect substrates for phosphorylation/activation by NPM‐ALK, which is the most common genetic heterogeneity in ALCL. Phosphorylated VAV activates the Rho family of GTPases (RAC, RHO, and CDC42). This results in changes in actin filament depolymerization and the loss of cell‐matrix adhesion, which contributes to the unusual sinusoidal growth pattern in this lymphoma.^2^ Lymphomatous infiltration of the alveoli may result in interlobular septal thickening and granulomatous consolidation on chest CT, and this may mimic the appearance of pneumonia or miliary tuberculosis. The presentation may also be similar to pulmonary lymphangitic carcinomatosis. Sinusoidal growth and perivascular distribution in the lymph nodes, characteristic of ALCL, can be found in approximately 75% of cases, and the clusters of large cells congesting the sinuses may mimic a metastatic carcinoma.^3^ Clinicians should perform biopsies to facilitate earlier initiation of the required treatment, prompt diagnosis and treatment to improve prognosis.

## CONFLICT OF INTEREST

All authors declare no conflicts of interest.

## AUTHOR CONTRIBUTIONS

SS and YT: participated in the management of this patient as well as in the preparation and edition of this manuscript. Both authors: read and approved the final manuscript.

## INFORMED CONSENT

Written informed consent was obtained from the patient.
